# Targeted Temperature Management Using Esophageal Cooling

**DOI:** 10.1089/ther.2022.0033

**Published:** 2022-11-25

**Authors:** Cedar Morrow Anderson, Christopher Joseph, Rick Fisher, Donald Berry, J. Brad Diestelhorst, Christine Kulstad, Marvin Wayne

**Affiliations:** ^1^ Intensive Care Unit, PeaceHealth St. Joseph Medical Center, Bellingham, Washington, USA.; ^2^ Department of Emergency Medicine, University of Texas Southwestern Medical Center, Dallas, Texas, USA.; ^3^ Emergency Medical Services, Attending, PeaceHealth St. Joseph Medical Center, University of Washington, Bellingham, Washington, USA.

**Keywords:** esophageal cooling, targeted temperature management, therapeutic hypothermia

## Abstract

Although specific temperature targets are debated, targeted temperature management (TTM) is a common treatment for postcardiac arrest patients. However, consistently implementing a TTM protocol is challenging, especially in a community hospital. Often, the protocols described in the literature include labor- and cost-intensive methods that are not feasible or sustainable in many health care settings. Esophageal temperature management (ETM) is a TTM method that can be easily utilized alone or combined with surface methods. We sought to evaluate ETM in a cohort of patients treated with TTM after cardiac arrest. Chart reviews were conducted of all patients treated with ETM after cardiac arrest at our community medical center. Initial patient temperature, time to target, supplemental methods (water blankets, chest wraps, or head wraps), and patient survival were extracted for analysis. A total of 54 patients were treated from August 2016 to November 2018; 30 received ETM only, 22 received supplemental cooling, and 2 had treatment discontinued before reaching target due to recovery. Target temperatures ranged from 32°C to 36°C, depending on provider preference. The median time to target temperature for the entire cohort was 219 minutes (interquartile range [IQR] 81–415). For the cohorts without, and with, supplemental cooling modalities, the median time to attain target temperature was 128 minutes (IQR 71–334), and 285 minutes (IQR 204–660), respectively. Survival to intensive care unit discharge was 51.9% for the entire cohort. Survivors exhibited longer times to achieve goal temperature (median 180 minutes in nonsurvivors vs. 255 minutes in survivors). ETM attains target temperature at a rate consistent with current guidelines and with similar performance to alternative modalities. As in other studies, surviving patients required longer times to reach target temperature.

## Introduction

Targeted temperature management (TTM) is a common treatment for postcardiac arrest patients (Neumar et al, 2015). However, consistently implementing a TTM protocol is challenging, especially in a community hospital. Often, the protocols, described in the literature, include labor- and cost-intensive methods that are not feasible or sustainable depending on the resources of the health care setting. For example, the intravascular cooling methods often outperform surface methods in terms of time to target temperature (Deye et al, 2015; Finley Caulfield et al, 2011; Gillies et al, 2010; Glover et al, 2016; Oh et al, 2015; Rosman et al, 2018; Wolff et al, 2009).

However, because intravascular cooling is physician (or advanced provider) initiated and introduces risks, including catheter-associated bloodstream infection (CLABSI), deep venous thrombosis (DVT), and pulmonary embolism (PE) (Gierman et al, 2013) (Gillon et al, 2015; Maze et al, 2014; Reccius et al, 2015; Wang et al, 2018), it may not be an ideal choice for some organizations. Surface devices can be deployed by a wider range of clinicians, such as nurses, and avoid CLABSI and reduce the risks of DVT and PE, but increase the incidence of shivering, which in turn impacts temperature maintenance and adds to the overall cost of care (Khan et al, 2018; van Zanten and Polderman, 2009).

Esophageal temperature management (ETM), a relatively newer TTM method, offers minimally invasive core access. ETM uses a device that is placed in the esophagus, in a similar manner to an orogastric tube. The device connects to an external water blanket heat exchanger, which provides cold or warm water in a closed circuit, thereby transferring heat across the patient's core. This approach avoids the risks of sterile intravascular placement and the challenges of transferring heat across the skin. Compatible heat exchangers can be used simultaneously with water blankets, offering additional cost-effectiveness and ease of implementation. A formal analysis of common performance metrics, such as time to target temperature, in a community hospital setting has not previously been performed. Thus, this study aimed to evaluate ETM performance, used alone or combined with surface methods, in a cohort of patients who received TTM after cardiac arrest.

## Methods

### Study design

This study was approved by PeaceHealth IRB (PHIRB 1085850-3) as a retrospective review of all patients treated with an ETM device (EnsoETM ECD02A; Attune Medical, Chicago, IL) for TTM after cardiac arrest in a single institution. All patients had attained return of spontaneous circulation after cardiac arrest from any rhythm, and were cooled according to local hospital protocol, using a Cincinnati Sub-Zero Blanketrol III Hyper-Hypothermia System (Gentherm Medical, Cincinnati, OH) connected to the ETM device. Additional surface wraps were added to patients at the discretion of the treating clinicians, and these were added to the same heat exchanger powering the ETM device, since no change otherwise is required for the heat exchanger already in operation, and the heat exchanger is able to accommodate a total of three devices in parallel simultaneously.

### Cooling protocol

The protocol at this site is to utilize the servo-control mode, which utilizes feedback control from current patient temperature to determine circulating water temperature. This in turn automatically adjusts water temperature without need for input from the treating clinicians. In general, supplemental cooling was initiated in patients in whom the cooling rate was felt to be suboptimal, which in general, as in most cases of clinical decision-making, was a decision made by treating practitioners at the patient's bedside. Regardless, no changes to target temperature were made in response to the use of supplemental cooling. No use of iced saline occurred in general in our intensive care unit (ICU) or in the prehospital system serving our hospital.

Body temperature was recorded hourly in general, utilizing Foley temperature sensor catheters. The hospital shivering protocol utilizes a variation of the Columbia shivering protocol, as described elsewhere (Jain et al, 2018). In general, cooling at our hospital is initiated upon arrival at the hospital, at the initial site of entry (emergency department or direct to ICU). In the case that patients went to receive percutaneous coronary intervention (PCI), cooling continued uninterrupted, since placement of the ETM device does not delay transport to the cath lab, and the presence of the device does not interfere with performance of PCI.

### Data collection

Charts were identified through a quality-control log of patients treated with temperature management. Details of treatment, including device used, temperature targeted, and time to target temperature were extracted by one study author (R.F.) and reviewed in cases of uncertainty by a group of other authors (C.M.A., D.B., J.B.D., and M.W.). Patient starting temperature, target temperature, and times of start and attainment of goal temperature were collected.

### Statistical analysis

Data are reported with descriptive statistics, utilizing parametric or nonparametric measures where appropriate.

## Results

A total of 54 patients were treated with ETM over the evaluation period of August 2016–November 2018. Median patient age was 63.5 years, with an interquartile range (IQR) of 41.8–70.3 years, and 52% were male. Median patient body mass index (BMI) was 27.9 (IQR 25–34.8). Presenting rhythm on EMS arrival was 19% ventricular fibrillation, 20% asystole or pulseless electrical activity, and the remainder unknown. Of these 54 patients, 30 had ETM only, 22 had supplemental, and 2 recovered and had treatment discontinued before reaching the target temperature. Target temperatures ranged from 32°C to 36°C, depending on clinician preference, with a range typically specified spanning 2°C–3°C (e.g., 34–36°C or 33–35°C).

The median time to attain target temperature for the entire cohort was 219 minutes (IQR 81–415). Patients treated with ETM only had a median time to target of 128 minutes (IQR 71–334) and patients who received supplemental surface cooling had a median time to target temperature of 285 minutes (IQR 204–660). Survival to ICU discharge was 51.9% for the entire cohort, and survivors exhibited longer times to achieve goal temperature (median 180 minutes in nonsurvivors vs. 255 minutes in survivors).

**FIG. 1.**
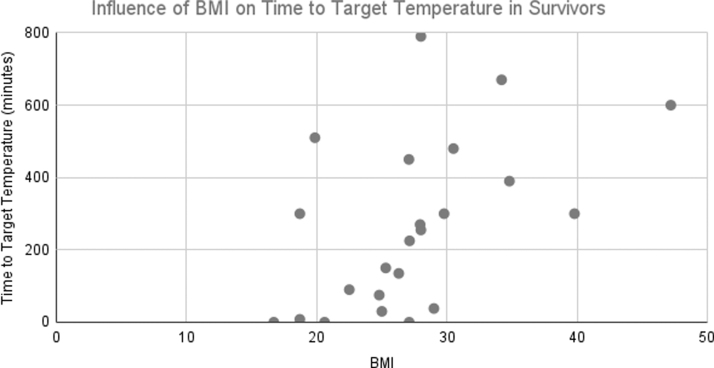
Influence of patient BMI on time to target temperature in patients surviving to ICU discharge. BMI, body mass index; ICU, intensive care unit.

**FIG. 2.**
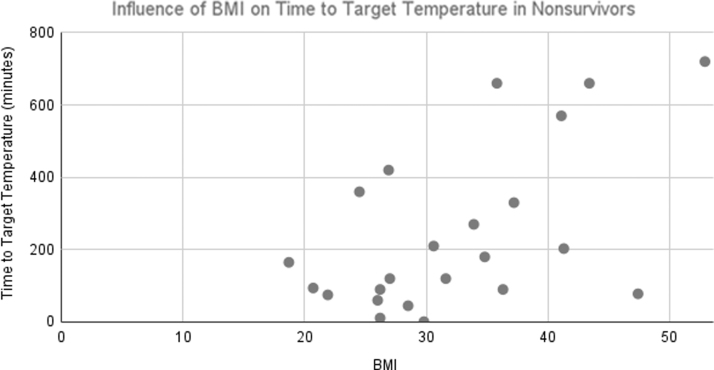
Influence of patient BMI on time to target temperature in patients not surviving to ICU discharge.

Patient BMI had an influence on time to target temperature, as seen in prior studies (Leary et al, 2014). Analysis of time to target temperature as a function of patient BMI is shown in Figure 1 (for survivors to ICU discharge) and Figure 2 (for patients not surviving to discharge from the ICU). As is evident from these plots, overall BMI did not differ significantly between survivors and nonsurvivors; however, times to goal temperature are notably greater in survivors. Of the 27 surviving patients, 11 received supplemental cooling, and in the 25 patients not receiving cooling, 11 received supplemental cooling (*p* = NS).

## Discussion

This is the largest study to date characterizing time to target temperature using ETM after cardiac arrest in a community hospital setting. An earlier study in a community hospital setting found trends toward improved neurological outcome in patients treated with hypothermia, but utilized more physician-driven protocols using intravascular cooling devices (Kulstad et al, 2010). A larger multi-institution survey that included 94 patients concluded that the implementation of TTM protocols across the country is extremely varied with no universally accepted treatment (Joseph et al, 2012). Our study suggests that utilization of a nursing-driven protocol with ETM as the modality of cooling may provide a straightforward solution to TTM objectives.

The aim of the study was to determine cooling rate in a large cohort of patients treated at a single institution. Although the study was not designed to make a direct comparison between esophageal cooling and other modalities, the data suggest that the cooling rate is comparable with other methods. A review of independent published data on alternative cooling methods used in postcardiac arrest TTM shows median times to target temperature ranging from 130 minutes to well >600 minutes (Table 1).

**Table 1.** Literature Summary of Targeted Temperature Management Methods in Pre-Hospital Cardiac Arrest Patients

Method	n	Target temperature	Time to target	References
Endovascular Femoral	203	33°C	426 (342–606) m^a^	Deye et al (2015)
Basic external cooling	197		600 (444–870) m	
Intravascular	240	33°C	210 [IQR 180] m	Glover et al (2016)
Surface	694		240 [IQR 180] m	
Intravascular	224	32–34°C	211.1 ± 12.9 m	Oh et al (2015)
Surface	559		240.1 ± 11.8 m	
Hydrogel surface pad^b^	69	32–34°C	166.8–480 m	Jarrah et al (2011)
Intravascular	26	32–34°C	240 (IQR 168–372) m	Finley Caulfield et al (2011)
Surface	15		270 (IQR 180–390) m	
Endovascular	34	32–34°C	240 m	Rosman et al (2018)
Traditional			360 m	
Intravascular	42	32–34°C	312 ± 198 m	Gillies et al (2010)
Surface	41		366 ± 288 m	
Intravascular	49^c^	33.0°C	410 (IQR 271–544) m	Wolff et al (2009)
Hydrogel surface pad	34	32–34°C	190 (IQR 135–155) m^a^	Heard et al (2010)
Standard surface	30		244 (IQR 180–300) m	
Intravascular	50	33.0°C	89 (IQR 52–155) m	Sawyer et al (2019)
Esophageal	17	32–34°C	240 m	Goury et al (2017)

^a^ Statistically significant.

^b^ Cooling adjuncts (ice packs and cold fluids) were added to expedite the cooling process in two cases.

^c^ Target temperature was not reached in 11 patients.

IQR, interquartile range; m, minutes.

Some patients arrived at lower core temperatures than others, allowing them to reach target more quickly, whereas others took much longer to cool and required supplemental cooling through head wraps, chest wraps, or blankets, to achieve target temperature. Although this retrospective study cannot identify the specific reasons that clinicians chose to utilize additional cooling modalities for particular patients, our study found that survivors exhibited longer times to achieve goal temperature, a finding similar to other reports in which a quicker time to target temperature is associated with worse outcome (Benz-Woerner et al, 2011; Haugk et al, 2011; Perman et al, 2014; Uber et al, 2018). In a study of 203 patients, nonsurvivors generated less heat than survivors and reached target temperature sooner (median 2.2 hours [IQR, 1.5–3.8 hours] vs. median 3.6 hours [IQR, 2.0–5.0 hours]; *p* = 0.01) (Uber et al, 2018).

A study of 177 patients found that median time to target temperature was shorter among nonsurvivors (200 [IQR 25–363] minutes) than survivors (270 [IQR 158–375] minutes, *p* = 0.03) (Benz-Woerner et al, 2011). A study of 321 patients found that time to target temperature in the “good outcome” (CPC 1–2) cohort was 237 (IQR 142–361) minutes compared with 180 (IQR 100–276) minutes in the “poor outcome” (CPC 3–5) cohort (*p* = 0.004) (Perman et al, 2014). A study of 588 patients found that time to target temperature was 209 minutes (IQR 130–302) in patients with favorable neurological outcomes compared with 158 minutes (IQR 101–230) (*p* < 0.01) in patients with unfavorable neurological outcomes (Haugk et al, 2011). The hypothesis for these differences has been that those who cool more quickly have lost the ability to thermoregulate, which may represent more severe brain injury, and thus, worse outcomes, in this subset of patients (Uber et al, 2018).

The TTM trial showed no difference in survival or survival with good neurological outcome when comparing 33°C and 36°C as a goal temperature (Nielsen et al, 2013). The TTM2 trial compared hypothermia with a goal temperature of 33°C to normothermia and again found no difference in the results (Dankiewicz et al, 2021), which can raise questions about the relevance of this data. However, in this trial patients in the normothermia group had their temperature actively managed when it rose above 37.8°C or higher, which occurred in 46% of their patients.

In addition, the external validity of these findings to a patient population with less frequent bystander cardiopulmonary resuscitation, or a lower proportion of shockable rhythms remains uncertain (Behringer et al, 2021). The ongoing Influence of Cooling Duration on Efficacy in Cardiac Arrest Patients (ICECAP) trial (NCT04217551) will better determine if the duration of cooling affects outcome among patients after cardiac arrest. At this time, temperature management is still the standard of care in patients after cardiac arrest. The ETM offered effective cooling, and was feasible for regular use in a community hospital setting. Further study is warranted to determine if its placement method decreases complication rates or patient care workload.

### Limitations

This study is limited by its retrospective nature; however, we utilized endpoints recorded in the medical record that are generally reliable and unlikely to be systematically biased. Although an inverse association was found between survival and cooling rate, no causation can be attributed. In addition, it was performed at a single community hospital site, which may limit the generalizability of our results. For this initial investigation, we focused on a few key outcomes and demographic variables, including patient age, gender, BMI, and initial cardiac rhythm when available. As such, for this initial report, we do not have a full complement of Utstein variables to report. Likewise, we do not have functional outcome data at this point, and are thus relegated to the surrogate of survival to ICU discharge.

## Conclusion

ETM attains target temperature at a rate consistent with current guidelines and with similar performance to alternative modalities. As in other studies, surviving patients required longer times to reach target temperature.
